# Dentoalveolar Abscess Caused by Pericoronitis of an Erupting First Molar

**DOI:** 10.3390/diagnostics15121531

**Published:** 2025-06-16

**Authors:** Kana Kawashima, Masashi Ogawa, Meiko Tachikake, Yuto Shoji, Tatsuya Akitomo, Ryota Nomura

**Affiliations:** Department of Pediatric Dentistry, Graduate School of Biomedical and Health Sciences, Hiroshima University, Hiroshima 734-8553, Japan; kanana25@hiroshima-u.ac.jp (K.K.); caries0@hiroshima-u.ac.jp (M.O.); meikosan@hiroshima-u.ac.jp (M.T.); shouji12@hiroshima-u.ac.jp (Y.S.); rnomura@hiroshima-u.ac.jp (R.N.)

**Keywords:** dentoalveolar abscess, pericoronitis, first molar, pediatric dentistry

## Abstract

**Background**: Pericoronitis is defined as inflammation of the soft tissues around the crown of an erupting tooth or a tooth with incomplete eruption, most commonly during eruption of the third molars. Pediatric dentists frequently encounter pericoronitis of the first molar, most of which resolve spontaneously. We describe the case of a 7-year-old girl who was referred to our hospital with intractable swelling in the right buccal region. **Case Presentation**: Intraoral examination showed an erupting right mandibular first molar and facial examination revealed swelling and an accumulation of pus in the cheek region. Radiographic examination revealed no pathological findings; therefore, it was diagnosed as a cheek abscess, and the region was incised that day. However, the symptoms recurred 3 weeks later, and cone-beam computed tomography detected a bone defect in the right mandibular first molar region, confirming a diagnosis of dentoalveolar abscess caused by pericoronitis of the first molar. The swelling resolved after incision of the abscess, and bone recovery was confirmed by X-ray in the follow-up period. **Conclusions**: Erupting first molars is at risk of pericoronitis, which may sometimes progress to a dentoalveolar abscess. Dental professionals should be alert to this possibility and should advise pediatric patients and their guardians to maintain good oral hygiene around erupting molars.

**Figure 1:**
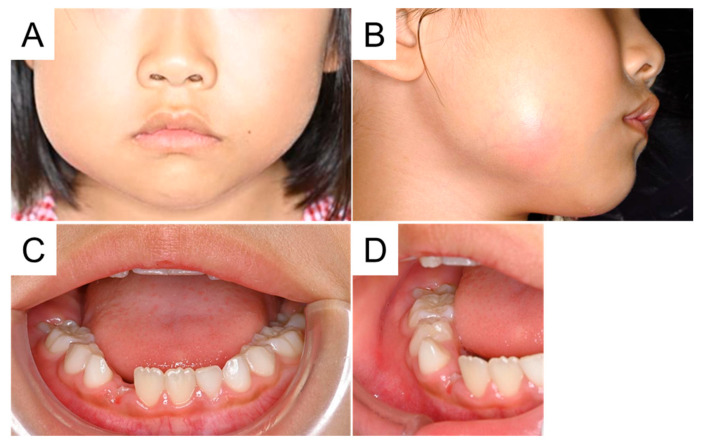
Facial and intraoral photographs at the initial visit. A girl aged 7 years and 3 months was referred to our hospital with a chief complaint of swelling in the right cheek region. She had felt pain in this region a month earlier and was prescribed antibiotics, which alleviated the symptoms. Ten days before her presentation, the swelling recurred and did not improve with antibiotics. She experienced trismus and pain; therefore, she visited the pediatric department of a general hospital, where ultrasonography detected an accumulation of pus in the right cheek region. The doctor determined that a surgical approach was required, so she was referred to our hospital. Her right cheek was swollen and painful to the touch (**A**,**B**). Her maximal mouth opening was 1.5 fingers wide and her body temperature was 36.9 °C, indicating a slight fever. Intraoral examination detected an erupting right mandibular first molar surrounded by painful gingiva (**C**,**D**). There was neither percussion pain nor drainage. The patient had no relevant medical and family history.

**Figure 2:**
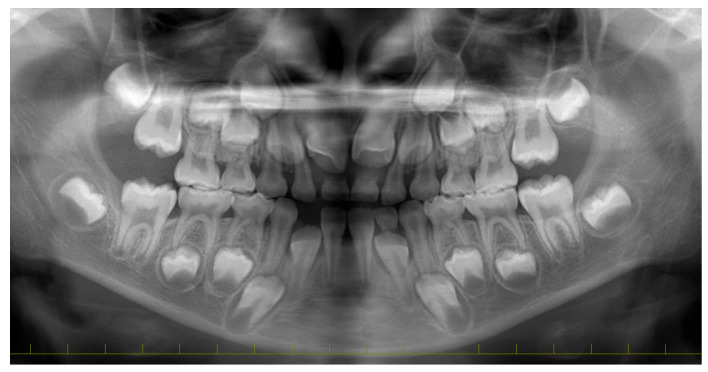
Panoramic examination revealed no pathological findings. However, a blood test revealed that the leukocyte count was 10.76 × 10^3^/µL and the C-reactive protein level was 0.43 mg/dL, suggesting inflammation. Although the source of the infection was unclear, it was diagnosed as a cheek abscess on the basis of the clinical symptoms. In collaboration with the Department of Oral Surgery, an incision was made in the buccal gingiva of the right mandibular primary molars, and the abscess was accessed and drained. Antibiotics (amoxicillin hydrate) and painkillers (acetaminophen) were prescribed.

**Figure 3:**
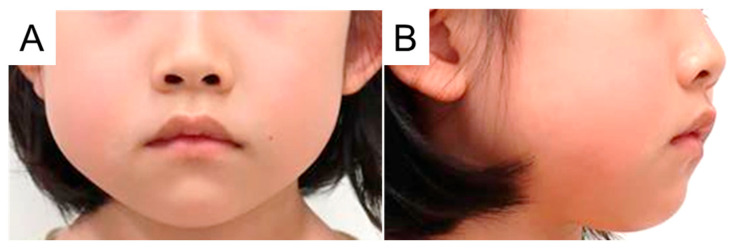
Facial photographs 4 days after incision of the abscess. This surgical approach enabled the patient to open her mouth. The swelling and pain in her cheeks also improved (**A**,**B**). We considered that an infection from the periodontal pocket of the first molar had caused the cheek abscess and continued with toothbrushing instructions.

**Figure 4:**
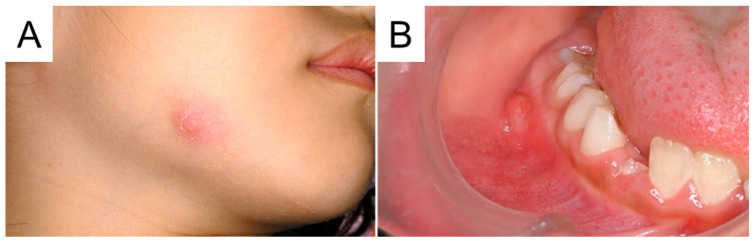
The cheek abscess recurred 3 weeks later when the patient bumped her cheek. There was no spontaneous pain, fever, or trismus; however, 15 mm of swelling and redness were observed at the inferior border of the mandible. Additionally, an intraoral abscess was found near the right mandibular primary second molar (**A**,**B**). Cone-beam computed tomography (CBCT) was obtained for a more detailed examination.

**Figure 5:**
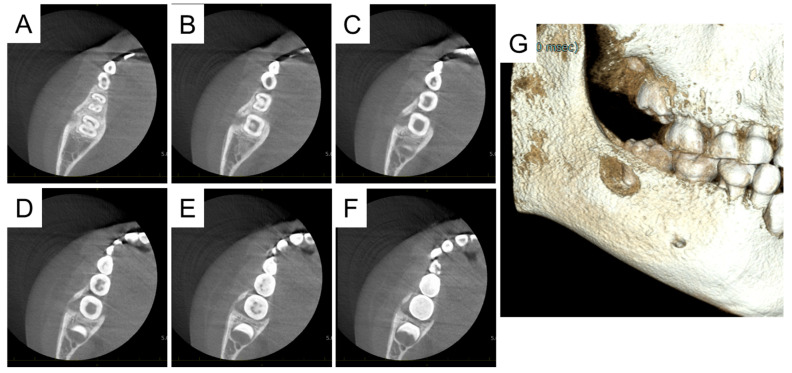
CBCT revealed an alveolar bone defect around the right mandibular first molar, confirming the diagnosis of a dentoalveolar abscess (**A**–**G**). In cooperation with the oral surgery department, surgical procedures were performed the next day. The gingival sulcus was incised, and the thinned alveolar bone and surrounding granulation tissue were removed. Antibiotic-containing gauze was placed, and the wound was sutured. Antibiotics and painkillers were prescribed, and the gauze was removed 3 days later. The pain was under control without the use of painkillers, and the redness, induration, and swelling were showing signs of amelioration. No purulent discharge was observed from the wound.

**Figure 6:**
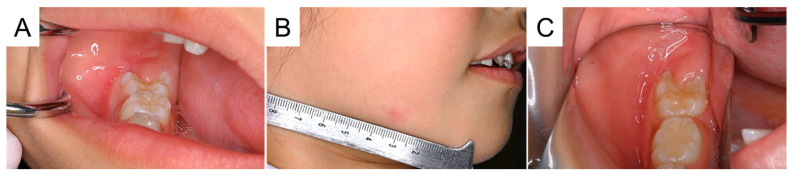
With continued regular irrigation of gingival pocket in mandibular first molar, the drainage and swelling gradually improved. At the age of 7 years and 5 months, there was no pain in the first molar region (**A**). The redness on the patient’s cheeks had gradually improved by the age of 7 years and 11 months (**B**). At the first visit, most of the crown of the first molar was covered by the gingival tissue. At that point, the abscess was far from the gums, and operculectomy with high invasion was not performed. Although it was still covered by gingival tissue at the age of 7 years and 11 months, an additional surgical approach was not required because there was no inflammation (**C**). CBCT was performed to confirm the state of bone formation and healing.

**Figure 7:**
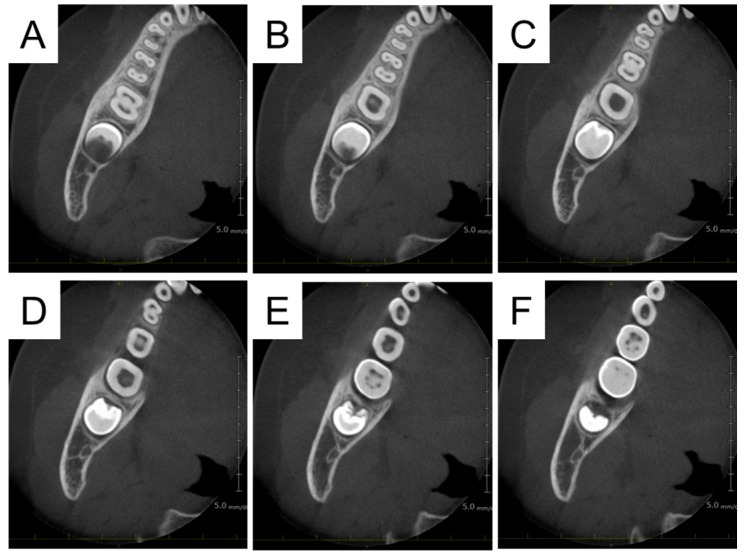
CBCT images at the age of 7 years and 11 months. The bone defect on the buccal side of the first molar had disappeared, and the continuity of the alveolar bone was confirmed, suggesting that the dentoalveolar abscess had completely healed (**A**–**F**).

**Figure 8:**
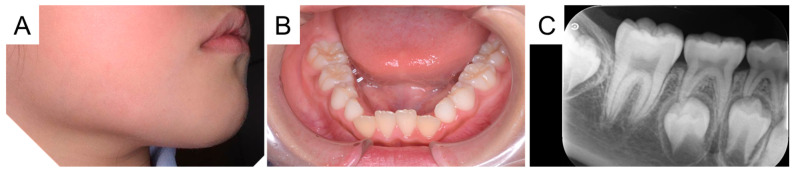
Facial and intraoral photographs at the age of 8 years and 10 months. The redness and swelling in the right cheek had completely healed and were no longer noticeable (**A**). The intraoral photograph showed the complete eruption of the right mandibular first molar, and periapical radiography confirmed the absence of pathology (**B**,**C**). The patient is currently continuing follow-up with the family dentist. In childhood, many children report discomfort during the eruption of the mandibular first molars, the pain is transient and occurs only when the new tooth crown is breaking through the mucosa [1]. However, slight inflammation may be observed in the epithelium surrounding the crown of the erupting new permanent tooth [1]. Pericoronitis is defined as inflammation of the soft tissues around the crown of an erupting tooth or a tooth with incomplete eruption [2]. Although it can occur with any erupting tooth, lower third molars are affected most frequently [2]. Signs of acute pericoronitis include swelling, constant trauma to the inflamed gingiva, exacerbated halitosis, purulent discharge, or systemic signs such as regional lymphadenopathy and low-grade fever [3]. In severe cases, the first line of treatment is the prescription of painkillers and antibiotics, and surgical extraction of the involved molar after the acute infection has been controlled [3]. A dentoalveolar abscess is a serious complication that may arise from untreated dental caries, periodontal disease, pericoronitis, or facial fractures, causing alveolar bone resorption or even tooth loss [4,5]. In the present case, although the mandibular right first molar was erupting in the oral cavity, panoramic examination revealed no pathological findings, and we initially diagnosed it as a cheek abscess. Surgical approaches improved the swelling in the buccal region; however, the swelling recurred 3 weeks later and CBCT detected a bone defect around the erupting tooth, confirming the diagnosis of a dentoalveolar abscess that had progressed from pericoronitis. To our knowledge, this is the first report of a dentoalveolar abscess caused by pericoronitis of a first molar during eruption. Because pericoronitis results from an overgrowth of bacteria in the hard-to-clean areas that form during tooth eruption, good oral hygiene is crucial in preventing pericoronitis [6]. Permanent mandibular first molars erupt at the age of 6.28 ± 0.74 years in Japanese females; however, children under the age of 9 years are usually considered to lack the developmental skills needed to brush their own teeth [7,8]. Thus, erupting first molars is at a high risk not only of dental caries but also of pericoronitis. Dental professionals often encounter pericoronitis of first molars in clinical pediatric dentistry, and most of them disappear naturally as the tooth eruption or improves with gingivoplasty. As in this case, it is rare for a tooth with no obvious problems other than pericoronitis to develop an alveolar abscess. Although the cause is unknown at this time, dental professionals should be aware that pericoronitis in first molars may progress to a dentoalveolar abscess. Our first diagnosis was cheek abscess without dental origin because of no pathological finding in the panoramic examination. Although it is unclear whether a bone defect existed in the first molar region at the initial visit, if CBCT had detected it at an early stage, the diagnosis and approach would have been different. The basic principles of radiation protection should always be kept in mind when using CBCT in children, and its use should be justified only in cases where conventional radiography fails to provide relevant information [9]. In the present case, the bone defect was first detected by CBCT despite no pathological findings in the initial radiographic examination. This report highlights the importance of detailed examinations by CBCT when swelling is observed in the maxillofacial region. In addition, toothbrushing by parents is associated with better oral hygiene in young children. Regular dental visits, combined with proper oral hygiene education, also lead to oral disease prevention [10]. Dental professionals should provide the parents with the importance of regular dental visits and appropriate oral health education to all children, considering each age group, which leads to the prevention of not only dental caries but also pericoronitis. Conditions that may account for acute facial swelling accompanied by inflammation include lymphadenitis, sinusitis, odontogenic infection, and abscess [11]. It is important for medical professionals to perform proper clinical decision-making from the differential diagnosis. In the present case, cheek swelling was caused by pericoronitis of the first molar, which was very rare case. Increasing the knowledge about the various causes of facial swelling will lead to better healthcare for pediatric patients.

## Data Availability

Data are contained within the article.
